# Supplementary material for: Targeted Profiling of Immunological Genes during Norovirus Replication in Human Intestinal Enteroids
Source: Viruses. 2021 Jan 21;13(2):155. doi: 10.3390/v13020155 (PMC7910953; doi:10.3390/v13020155)
Supplement: Supplementary file 1 [file viruses-13-00155-s001.pdf]

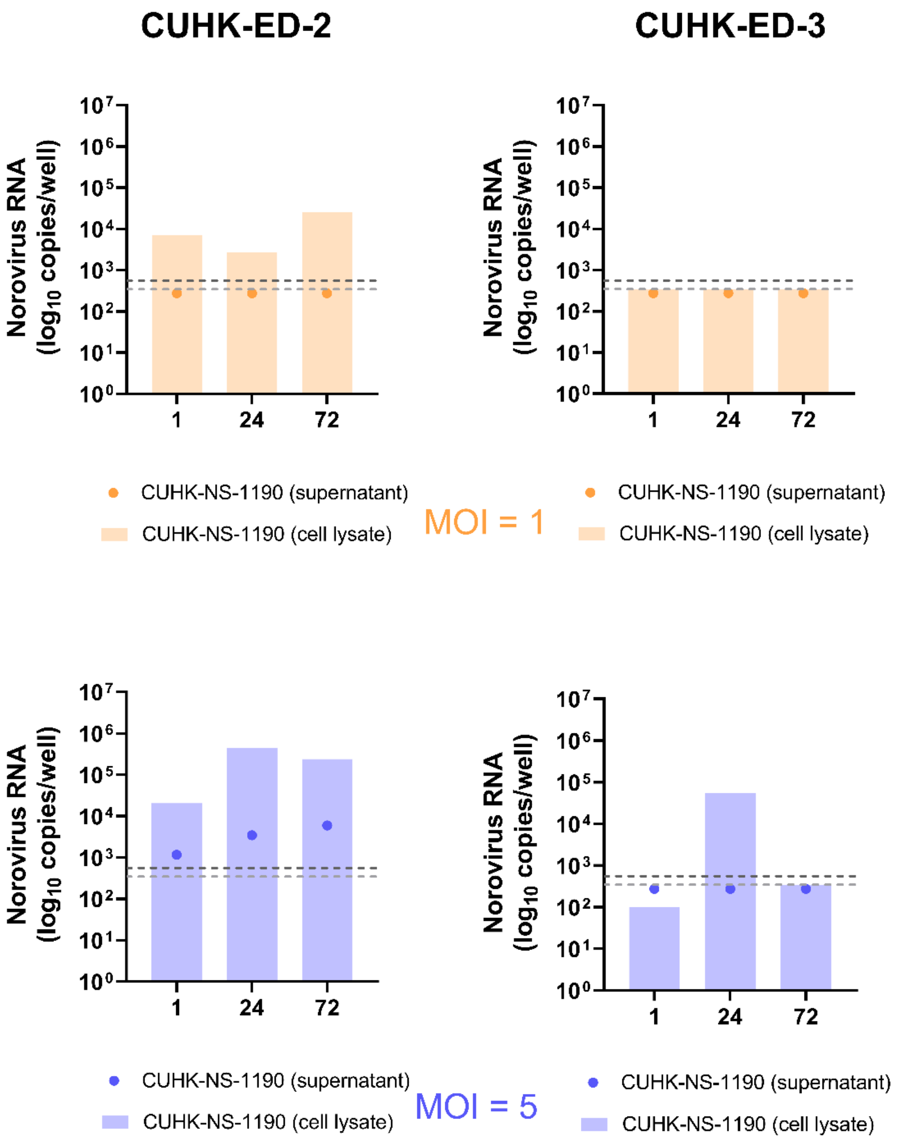

Figure S1. Monolayer of each human intestinal enteroids lines were inoculated at a multiplicity of infection of 1 and 5 (5.7 × 104 and 2.8 × 105 genome equivalents/well, respectively) with CUHK-NS-1190. Noroviral RNA was extracted from supernatant and cell lysates collected at 1, 24 and 72 hours post-inoculation (hpi), viral RNA copies per well were obtained by quantitative reverse transcription polymerase chain reaction (RT-qPCR). The horizontal grey and silver dotted line indicates the lower detection limit of RT-qPCR at 552 copies per well of supernatant and 342 copies per well of cell lysates, respectively.

Table S1. Cell markers used in quantification of gene expression of six intestinal epithelial cell types by TaqMan Gene Expression Assays.

Cell type	Target	Gene	Assay reference	Supplier
Stem cells	Leucin-rich-repeat-containing G-protein-couple receptor 5	LGR5	Hs00969422_m1	Thermo Fisher
Enterocytes	Sucrose isomaltase	SI	Hs00356112_m1	Thermo Fisher
Enteroendocrine cells	Chromogranin A	CHGA	Hs00900370_m1	Thermo Fisher
Goblet cells	Mucin 2	MUC2	Hs00159374_m1	Thermo Fisher
Paneth cells	Defensin 5	DEFA5	Hs00360716_m1	Thermo Fisher
Tuft cells	doublecortin like kinase 1	DCLK1	Hs00178027_m1	Thermo Fisher

Table S2. Multivariate linear regression analysis of gene expression of CXCL10 with cell-associated noroviral RNA level.

Coefficients[a]

Model		Unstandardized Coefficients		Standardized Coefficients	t	Sig.
		B	Std. Error	Beta		
1	(Constant)	53.321	144.361		.369	.716
	HIE_line	20.321	51.712	.097	.393	.699
	Strain	-57.902	37.835	-.277	-1.530	.142
	Time_point	-.849	.712	-.241	-1.192	.248
	Noroviral_RNA_fold_change_CP	1.009	.389	.686	2.590	.018

a. Dependent Variable: CXCL10_fold_change

Table S3. Multivariate linear regression analysis of gene expression of IFI44L with cell-associated noroviral RNA level.

Coefficients[a]

Model		Unstandardized Coefficients		Standardized Coefficients	t	Sig.
		B	Std. Error	Beta		
1	(Constant)	37.540	278.761		.135	.897
	HIE_line	58.300	99.856	.184	.584	.578
	Strain	-105.183	73.059	-.332	-1.440	.193
	Time_point	-1.738	1.375	-.325	-1.264	.247
	Noroviral_RNA_fold_change_CP	2.065	.752	.926	2.745	.029

a. Dependent Variable: IFI44L_fold_change

Table S4. Multivariate linear regression analysis of secretion of CXCL10 with supernatant and cell-associated noroviral RNA level.

Coefficients[a]

Model		Unstandardized Coefficients		Standardized Coefficients	t	Sig.
		B	Std. Error	Beta		
1	(Constant)	14.108	17.653		.799	.455
	HIE_line	4.610	6.125	.046	.753	.480
	Strain	-.212	4.113	-.002	-.052	.960
	Time_point	.089	.102	.053	.877	.414
	Noroviral_RNA_CP	-2.110E-5	.000	-.352	-5.407	.002
	Noroviral_RNA_SN	.001	.000	1.205	14.586	.000

a. Dependent Variable: CXCL10_ELISA
